# HIF isoforms have divergent effects on invasion, metastasis, metabolism and formation of lipid droplets

**DOI:** 10.18632/oncotarget.4612

**Published:** 2015-07-20

**Authors:** Tariq Shah, Balaji Krishnamachary, Flonne Wildes, Yelena Mironchik, Samata M. Kakkad, Desmond Jacob, Dmitri Artemov, Zaver M. Bhujwalla

**Affiliations:** ^1^ JHU ICMIC Program, Division of Cancer Imaging Research, The Russell H. Morgan Department of Radiology and Radiological Science, Baltimore, MD, USA; ^2^ Sidney Kimmel Comprehensive Cancer Center, The Johns Hopkins University School of Medicine, Baltimore, MD, USA

**Keywords:** HIF, hypoxia, invasion, metabolism, lipids

## Abstract

Cancer cells adapt to hypoxia by the stabilization of hypoxia inducible factor (HIF)-α isoforms that increase the transcription of several genes. Among the genes regulated by HIF are enzymes that play a role in invasion, metastasis and metabolism. We engineered triple (estrogen receptor/progesterone receptor/HER2/neu) negative, invasive MDA-MB-231 and SUM149 human breast cancer cells to silence the expression of HIF-1α, HIF-2α or both isoforms of HIF-α. We determined the metabolic consequences of HIF silencing and the ability of HIF-α silenced cells to invade and degrade the extracellular matrix (ECM) under carefully controlled normoxic and hypoxic conditions. We found that silencing HIF-1α alone was not sufficient to attenuate invasiveness in both MDA-MB-231 and SUM149 cell lines. Significantly reduced metastatic burden was observed in single (HIF-1α or HIF-2α) and double α-isoform silenced cells, with the reduction most evident when both HIF-1α and HIF-2α were silenced in MDA-MB-231 cells. HIF-2α played a major role in altering cell metabolism. Lipids and lipid droplets were significantly reduced in HIF-2α and double silenced MDA-MB-231 and SUM149 cells, implicating HIF in their regulation. In addition, lactate production and glucose consumption were reduced. These results suggest that *in vivo*, cells in or near hypoxic regions are likely to be more invasive. The data indicate that targeting HIF-1α alone is not sufficient to attenuate invasiveness, and that both HIF-1α and HIF-2α play a role in the metastatic cascade in these two cell lines.

## INTRODUCTION

Hypoxia arises as a result of the chaotic vasculature that forms in tumors [1, 2]. The adaptive response of cells to low oxygen tension is mediated through the hypoxia-inducible factor (HIF) [3]. HIF is a heterodimeric basic helix-loop-helix PAS (Per-ARNT-Sim) domain containing transcription factor that consists of a constitutively expressed β-subunit (HIF-β/ARNT) and one of three oxygen-regulated α-subunits, HIF-1α, HIF-2α and HIF-3α [4, 5]. The α-subunits are constitutively transcribed and translated, but are regulated at the protein level by oxygen-dependent hydroxylation of specific prolyl residues. These prolyl residues target the α-subunits for ubiquitination by the von Hippel-Lindau (VHL) protein-E3 ubiquitin ligase complex, and for subsequent proteasomal degradation. In addition, oxygen dependent asparagine hydroxylation in the C-terminal transactivation domain of the HIF-α subunits modulates their transcriptional activity. Cancer cells display an adaptive response to hypoxia through the binding of HIF to hypoxia response elements in the promoter region of target genes that results in increased transcription of these genes. HIF-regulated genes have been reported to promote angiogenesis, metabolism, proliferation, metastasis, and de-differentiation in cancer cells [3]. HIF-α subunits may also be stabilized in the absence of hypoxia by growth factor signaling pathways [6] such as activation of RAS and Src oncogenes, mutation in the tumor suppressor PTEN gene resulting in activation of HIF that is mediated through PI3K, and loss or inactivation of the tumor suppressor gene VHL [7]. The expression of both HIF-1α and HIF-2α are increased in several human tumors, including bladder, breast, colon, glial, hepatocellular, ovarian, pancreatic, prostate, and renal tumors [8]. In clinical specimens, elevated HIF expression has been correlated with poor prognosis [9–11]. HIF-1α activation has also been associated with resistance to endocrine and chemotherapies [12].

Metastasis, a primary cause of breast cancer mortality, occurs in a series of distinct steps that include tumor cell invasion, intravasation, extravasation, and proliferation, at distant sites. Activation of HIF regulated genes has been correlated with metastasis in multiple tumors and promotes metastasis [3]. The role of hypoxia in breast tumor pathology and as a significant indicator of poor prognosis is well established [13]. Several studies have linked higher HIF-1α levels to poor outcome in various patient subgroups of invasive breast cancer [11–13]. Tumors with poor prognosis, such as with estrogen and progesterone receptor (ER/PR)-negative status, have higher proportions of anoxic and hypoxic areas [11]. Basal like breast carcinomas show a stronger expression of known hypoxia related genes [14], as well as a higher glycolytic metabolism than tumors with luminal characteristics [15, 16].

While studies have focused on detecting HIF isoforms in various cancers, the role of individual isoforms in actively regulating extracellular matrix (ECM) degradation, invasion, metastasis, and lipid and glucose metabolism is relatively unexplored. Here we have investigated the role of HIF isoforms in these phenotypic characteristics of cancer using two triple (ER/PR/Her2/neu) negative breast cancer (TNBC) cell lines. The MDA-MB-231 human breast cancer cell line is an invasive and metastatic TNBC cell line that strongly expresses both HIF-1α and HIF-2α proteins under hypoxia [17]. SUM149 TNBC cells were derived from inflammatory ductal carcinoma of the breast and have been shown to express HIF-1α [18]; HIF-2α expression has not been investigated in these cells. We genetically engineered MDA-MB-231 cells and SUM-149 cells to express short hairpin RNA (shRNA) against either HIF-1α (231-HIF-1α shRNA, SUM-HIF-1α shRNA), HIF-2α (231-HIF-2α shRNA, SUM-HIF-2α shRNA) or both α isoforms (231-DS, SUM-DS) to silence the expression of HIF isoforms.

The invasiveness of MDA-MB-231 and SUM149 cells and their corresponding HIF isoform silenced sublines were investigated using a transwell chamber assay. Changes in glycolysis and the formation of lipid droplets were also investigated in both TNBC cell lines and their corresponding HIF isoform silenced sublines. Molecular validation of HIF silencing and characterization of pathways underlying the changes in metabolism was also performed.

We further investigated the ability of intact MDA-MB-231 cells and HIF isoform silenced sublines to invade and degrade the extracellular matrix (ECM) under normoxic or hypoxic conditions, and determined the changes in metabolism of these intact cells, in our magnetic resonance (MR) imaging and spectroscopy compatible cell perfusion assay. The metastatic burden established by these cells was characterized following intravenous injection of cells.

## RESULTS

### Characterization of genetically engineered MDA-MB-231 sublines

**Figure 1:**
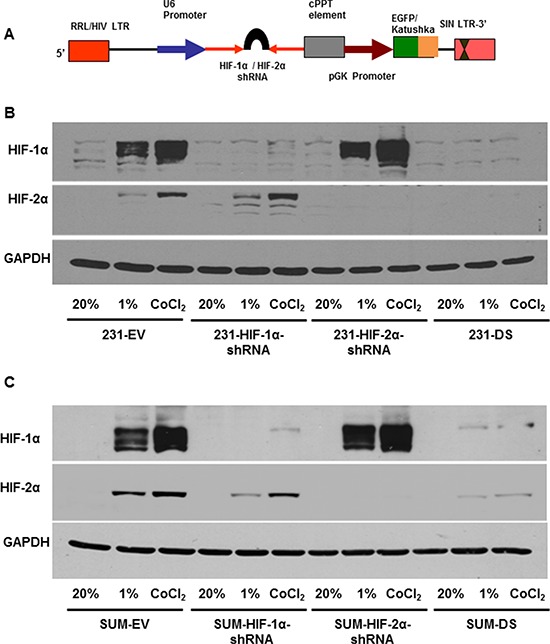
**A.** Schematic of vector construct used for generating HIF-α silenced cells with fluorescent reporters. Immunoblots showing HIF-1α and HIF-2α expression levels in HIF silenced and double silenced cells relative to double empty vector in **B.** MDA-MB-231 and **C.** SUM-149 cells, in response to 1% O_2_ and 200 μM CoCl_2_.

Elements of the lentiviral plasmid vector used for silencing MDA-MB-231 and SUM-149 cells are shown in Figure 1A. Whole cell protein extracts from genetically modified MDA-MB-231 and SUM-149 cells maintained under normoxia, hypoxia or treated with the hypoxia mimetic CoCl_2_ for 4–5 h were resolved on 7% SDS gel and immunoblotted against HIF-1α or HIF-2α specific antibody. Representative immunoblots shown in Figure 1B and 1C demonstrate reduced expression of HIF-1α and HIF-2α protein in HIF silenced cells as compared to cells expressing an empty vector (231-EV or SUM-EV). Both HIF-1α and HIF-2α proteins were stabilized under hypoxia in control MDA-MB-231 and SUM149 cells. Stabilization of HIF-1α or HIF-2α protein was not observed in HIF-1α shRNA and HIF-2α shRNA expressing breast cancer cells. 231-DS and SUM-DS cells showed reduced expression of both HIF-1α and HIF-2α under hypoxia.

### Double silenced cells show significant reduction in invasion

**Figure 2:**
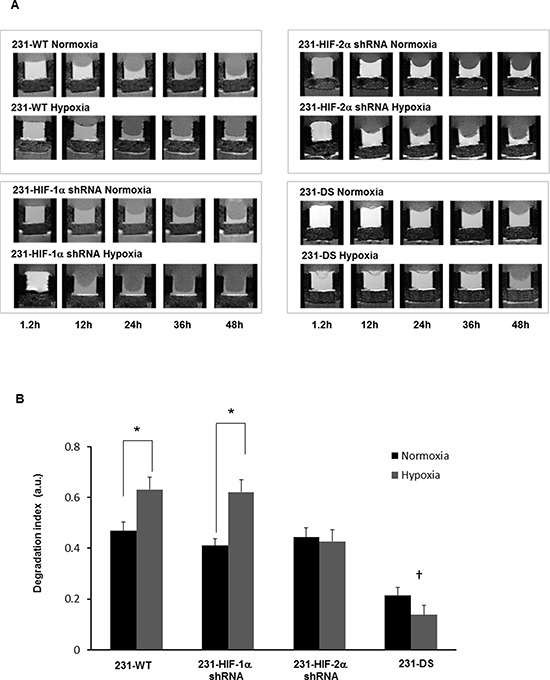
**A.** Representative T_1_-weighted ^1^H MR images zoomed to display the region with the ECM chamber, showing degradation of ECM gel by MDA-MB-231 sublines under normoxia and hypoxia at various time points. **B.** Degradation index estimated from ECM gel degradation at 24 h relative to the initial time point (*p < 0.05 between normoxia and hypoxia); (†*p* < 0.05 compared to parental or 231-HIF-1α or 231-HIF-2α shRNA cells under normoxia or hypoxia). 231-WT (*n* = 7 for normoxia and *n* = 6 for hypoxia, 231-HIF-1α shRNA (*n* = 7 for normoxia and *n* = 5 for hypoxia), 231-HIF-2α shRNA (*n* = 3 for normoxia and *n* = 3 for hypoxia), 231-DS (*n* = 5 for normoxia and *n* = 4 for hypoxia). Values represent Mean ± SE.

**Figure 3:**
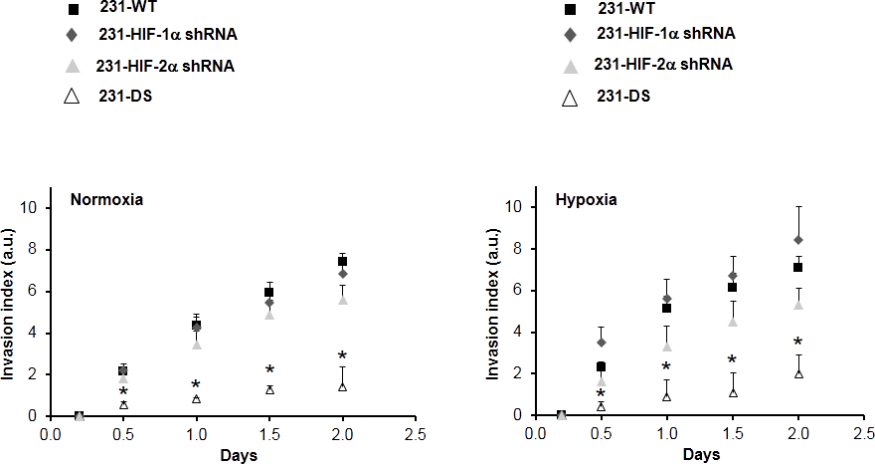
Invasion index *vs* time obtained from intracellular water signal over two days for MDA-MB-231 sublines under normoxia and hypoxia (*p < 0.05 double silenced *vs* all). Values represent Mean ± SE; 231-WT (*n* = 7 for normoxia and *n* = 6 for hypoxia), 231-HIF-1α shRNA (*n* = 7 for normoxia and *n* = 5 for hypoxia), 231-HIF-2α shRNA (*n* = 3 for normoxia and *n* = 3 for hypoxia), 231-DS (*n* = 3 for normoxia and *n* = 4 for hypoxia).

Representative T_1_-weighted ^1^H MR images demonstrating degradation of ECM gel by MDA-MB-231 wild type (231-WT) and 231-HIF-α shRNA cells under normoxia or hypoxia are shown in Figure 2A. The ECM gel was completely degraded by 231-WT cells at 48 h under normoxic conditions. Under hypoxic conditions, 231-WT cells completely degraded the ECM at an earlier time of 24 h. Both, 231-HIF-1α shRNA and 231-HIF-2α shRNA cells showed comparable matrigel degradation as parental MDA-MB-231 cells under normoxia. However, in response to hypoxia, HIF-2α shRNA cells did not show enhanced ECM degradation compared to parental and 231-HIF-1α shRNA cells. Double silenced cells showed significantly reduced degradation of ECM compared to parental cells under both normoxic and hypoxic conditions. These data are summarized for 24 h in Figure 2B and show a significant increase of the degradation index in parental and HIF-1α shRNA cells with hypoxia (*p* < 0.05) that was not observed in HIF-2α and double silenced cells. Double silenced cells also showed significant decrease of degradation even under normoxic conditions compared to parental cells (*p* < 0.05). Data showing changes in the invasion index with time are summarized in Figure 3. A significant reduction in the invasion index of 231-DS cells compared to parental and individual HIF isoform silenced cells (*p* < 0.05) was observed. No significant difference in the invasion index was observed between normoxic and hypoxic conditions for each MDA-MB-231 subline.

**Figure 4:**
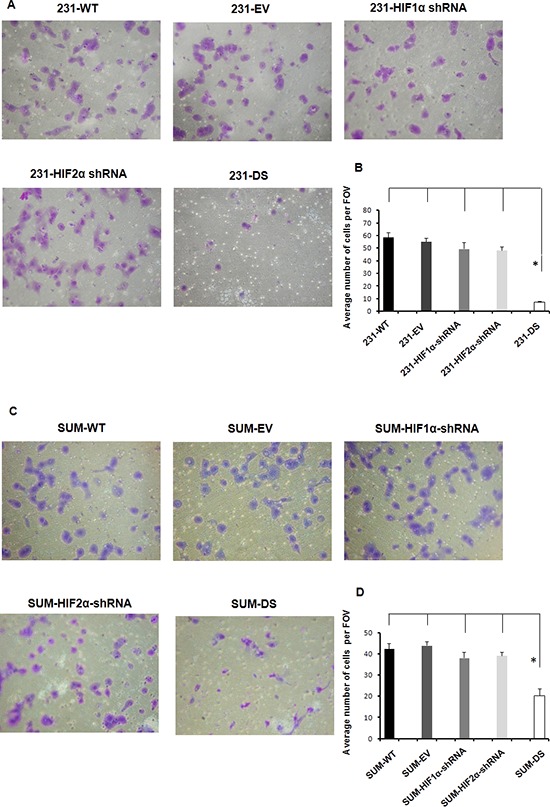
**A.** Representative images shown at 10X and **B.** quantitation of MDA-MB-231 sublines that invaded through a matrigel coated transwell chamber (*p < 0.05). **C.** Representative images shown at 10X and **D.** quantitation of SUM149 sublines that invaded through a matrigel coated transwell chamber (*p < 0.05). Values represent Mean ± SE with *n* = 4 for each group.

These data were further validated in MDA-MB-231 and SUM149 cells using the conventional transwell invasion assay. Figure 4 shows representative images (Figure 4A) and quantitation (Figure 4B) of MDA-MB-231 sublines that invaded through a matrigel coated transwell chamber. A significant reduction of cell invasion was only observed in double silenced cells (*p* < 0.05) consistent with data obtained using the perfusion assay. The importance of double silencing was further confirmed in SUM149 cells. The transwell invasion assay performed with SUM149 sublines showed a significant reduction in cell invasion (Figures 4C and 4D) only in the SUM-DS subline (*p* < 0.05).

### Metastatic burden was reduced in HIF silenced cells

**Figure 5:**
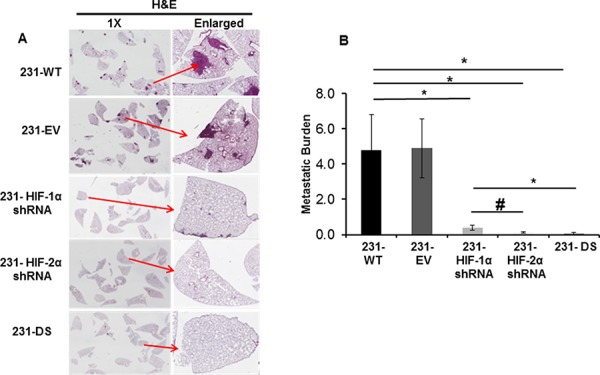
Metastatic burden following injection of 231-WT, 231-EV and 231-HIF shRNA cells through the tail vein. **A.** Representative images of H&E stained 5 mm thick lung sections acquired at 1X and enlarged. **B.** Quantitation of metastatic burden (*p < 0.05, #*p* < 0.08). Values represent Mean ± SE, *n* = 5 mice for each group.

To further understand the role of HIF-α isoforms in the extravasation and colonization of aggressive MDA-MB-231 breast cancer cells in the lung, histological analysis of lungs isolated from mice injected with 231-WT and the HIF-α sub lines was performed. As evident in the representative images in Figure 5A, silencing of single or both isoforms of HIF-α had a profound effect on reducing lung colonization by the cancer cells. A summary of metastatic burden shown in Figure 5B demonstrates the significant reduction (*p* < 0.05) of this parameter in lungs of mice injected with 231-HIF-1α shRNA, 231-HIF-2α shRNA, and 231-DS cells, compared to 231-WT and 231-EV cells. The largest decrease of metastatic burden was evident in the 231-DS cells.

### Altered metabolism in HIF silenced cells

**Figure 6:**
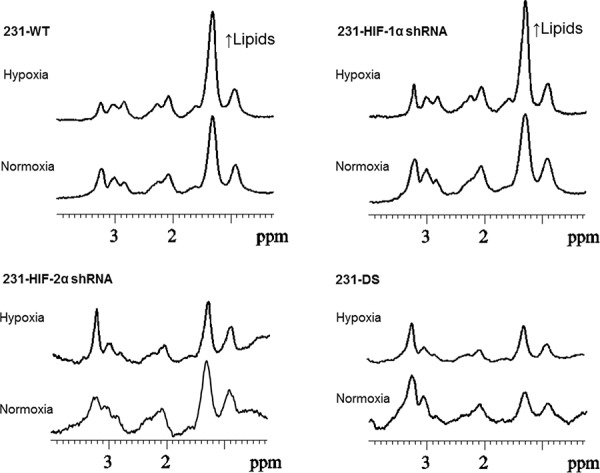
Representative ^1^H spectra obtained from MDA-MB-231 sublines maintained under normoxia and hypoxia for 36 h demonstrating changes in lipid signals under hypoxia.

**Figure 7:**
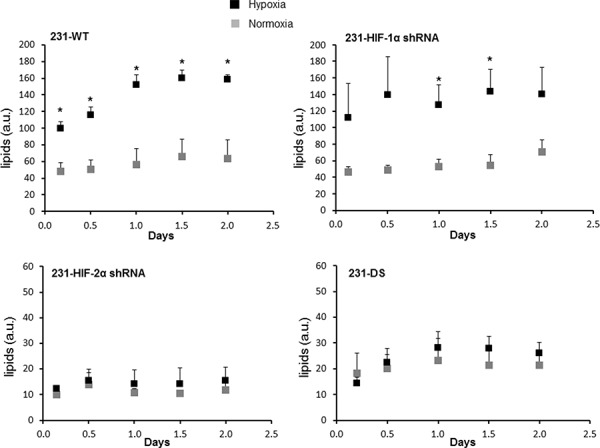
Quantification of lipid signal in arbitrary units from ^1^H MR spectra demonstrating differences in lipids (*p < 0.05). 231-WT (*n* = 7 for normoxia and *n* = 6 for hypoxia), 231-HIF-1α shRNA (*n* = 7 for normoxia and *n* = 5 for hypoxia), 231-HIF-2α shRNA (*n* = 3 for normoxia and *n* = 3 for hypoxia), 231-DS (*n* = 5 for normoxia and *n* = 4 for hypoxia).

Representative ^1^H MR spectra from the perfused cells under normoxia and hypoxia are displayed in Figure 6 and demonstrate the increase of the lipid signal in 231-WT and 231-HIF-1α-shRNA cells under hypoxia. 231-HIF-2α shRNA and 231-DS cells, on the other hand, had inherently low lipid signals that did not increase under hypoxia. Quantitative analyses of the lipid signals are shown in Figure 7 and demonstrate the significant increase of lipids in response to hypoxia in parental and HIF-1α shRNA cells (*p* < 0.05). Compared to parental cells, a significantly lower lipid level was observed in both HIF-2α shRNA and 231-DS cells (*p* < 0.05) that did not increase with hypoxia. There were no significant differences in other metabolites detected in the spectra.

**Figure 8:**
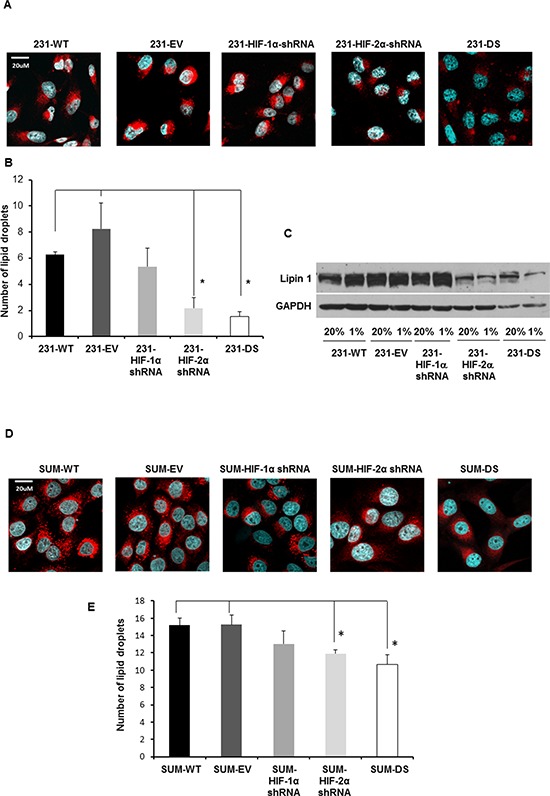
**A.** Representative confocal images acquired at 40X with lipid droplets visualized by Nile Red staining (red) and nuclei by DAPI staining (blue) in MDA-MB-231 sublines. **B.** Quantification of lipid droplets in MDA-MB-231 sublines (*p < 0.05). **C.** Immunoblot showing Lipin 1 expression under normoxia and hypoxia in MDA-MB-231 sublines. **D.** Representative confocal images acquired at 40X with lipid droplets visualized by Nile Red staining (red) and nuclei visualized by DAPI staining (blue) in SUM149 sublines. **E.** Quantification of lipid droplets in SUM149 sublines (*p < 0.05). Approximately 20–40 cells per FOV from six different fields of view were analyzed for each *n* value. Values represent Mean ± SE, *n* = 4.

To understand the reason underlying the significant increase of the lipid signal, immunocytochemistry for lipid droplets and immunoblot analysis for Lipin 1, was performed in MDA-MB-231 and SUM-149 sublines. Representative Nile Red stained images of lipid droplets for MDA-MB-231 cells and their sublines are shown in Figure 8A. Upon quantification of the lipid droplets (Figure 8B), it was apparent that significantly fewer lipid droplets were present in HIF-2α and double silenced cells compared to control cells (*p* < 0.05). As shown in the representative immunoblot for Lipin 1 in Figure 8C, Lipin 1 levels were higher in 231-WT, 231-EV and 231-HIF-1α shRNA cells compared to 231-HIF-2α shRNA and 231-DS cells. Unlike 231-WT, 231-EV and 231-HIF-1α shRNA cells, increased induction of Lipin 1 observed under hypoxia was not observed in 231-HIF-2α shRNA and 231-DS cells.

Representative Nile Red stained images of lipid droplets for SUM149 cells and their sublines are shown in Figure 8D. As in the MDA-MB-231 cells, quantification of the lipid droplets in SUM149 cells (Figure 8E), demonstrated significantly fewer lipid droplets in HIF-2α and double silenced cells compared to control cells (*p* < 0.05). Lipin 1 was not detected by immunoblotting in SUM149 cells.

**Figure 9:**
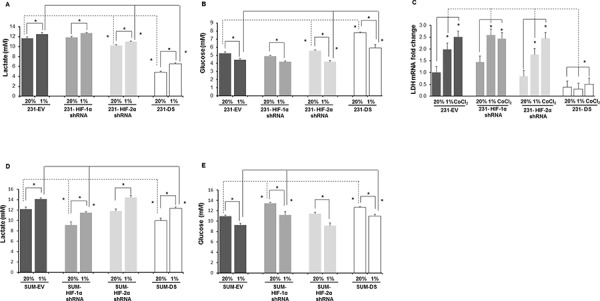
Quantification of **A.** lactate and **B.** glucose in the culture medium of MDA-MB-231 sublines after 72 h of normoxia, hypoxia or 200 μM CoCl_2_ treatment. **C.** LDHA mRNA levels in cells after 72 h of normoxia, hypoxia or 200 μM CoCl_2_ treatment. *p < 0.05, for the comparisons shown in the figure. 231-EV (*n* = 9 for normoxia and *n* = 9 for hypoxia), 231-HIF-1α shRNA (*n* = 3 for normoxia and *n* = 3 for hypoxia), 231-HIF-2α shRNA (*n* = 3 for normoxia and *n* = 3 for hypoxia), 231-DS (*n* = 3 for normoxia and *n* = 3 for hypoxia). Data from EV cells corresponding to the HIF silenced counterparts (3 for each subline) were pooled together for comparison. Quantification of **D.** lactate and **E.** glucose in the culture medium of SUM-149 sublines after 72 h of normoxia or hypoxia. SUM-EV (*n* = 3 for normoxia and *n* = 9 for hypoxia), SUM-HIF-1α shRNA (*n* = 3 for normoxia and *n* = 3 for hypoxia), SUM-HIF-2α shRNA (*n* = 3 for normoxia and *n* = 3 for hypoxia), SUM-DS (*n* = 3 for normoxia and *n* = 3 for hypoxia). *p < 0.05, for the comparisons shown in the figure. Values represent Mean ± SE.

To further characterize changes in metabolism we determined extracellular lactate and glucose concentrations in the culture medium of MDA-MB-231 and SUM149 sublines. Figure 9A shows the extracellular lactate concentration measured in culture medium of MDA-MB-231 cells and sublines after exposure to 72 h of hypoxia. A significant increase of lactate was observed in response to hypoxia in all the cells (*p* < 0.05). Significantly lower lactate concentrations were observed in HIF-2α and double silenced cells relative to the corresponding empty vector cells (*p* < 0.05) under normoxic conditions and hypoxic conditions. Complementing the lactate data, as shown in Figure 9B, glucose concentrations decreased in culture medium of MDA-MB-231 cells and sublines after exposure to 72 h of hypoxia (*p* < 0.05). Significantly higher glucose concentrations were observed in HIF-2α and double silenced cells relative to the corresponding empty vector cells (*p* < 0.05) under normoxic conditions and hypoxic conditions (*p* < 0.05). Figure 9C shows the fold changes in lactate dehydrogenase (LDH)-A mRNA levels in these cells quantitated by qRT-PCR under hypoxia and normoxia. Significant induction of LDHA mRNA was observed with hypoxia and CoCl_2_ treatment in empty vector, HIF-1α shRNA and HIF-2α shRNA cells compared to their respective normoxic counterparts (*p* < 0.05). However no induction of LDHA mRNA in response to hypoxia was observed in 231-DS cells. Double silenced cells showed a significant reduction of LDHA mRNA compared to the empty vector cells (*p* < 0.05).

Similar to MDA-MB-231 cells, SUM149 cells and their HIF-α isoform silenced sublines showed a significant increase of lactate in culture medium following exposure to 72 h of hypoxia (Figure 9D). Similar to the MDA-MB-231 cells, significantly lower lactate concentrations were observed in double silenced cells relative to the corresponding empty vector cells (*p* < 0.05) under normoxic conditions and hypoxic conditions. However, unlike MDA-MB-231 cells, in the case of SUM149, HIF-1α silenced cells showed a significantly lower lactate concentration compared to empty vector cells under normoxic and hypoxic conditions. These changes in lactate concentrations were reflected in corresponding changes in glucose concentrations (Figure 9E). Glucose concentrations decreased in culture medium of SUM149 cells and sublines after exposure to 72 h of hypoxia (*p* < 0.05). Significantly higher glucose concentrations were observed in HIF-1α and double silenced cells relative to the corresponding empty vector cells (*p* < 0.05) under normoxic conditions and hypoxic conditions (*p* < 0.05).

## DISCUSSION

Silencing HIF-1α and HIF-2α individually and together had divergent effects on invasion, metastasis, lipid droplets, and metabolism in the two TNBC cell lines investigated here. Silencing both isoforms resulted in the most significant changes in the pathways investigated in both cell lines including decrease of glycolysis under normoxia and hypoxia. Of the two isoforms, silencing HIF-2α was the more effective in reducing ECM degradation under hypoxia, decreasing experimental metastasis, and reducing lipids and lipid droplets following investigation in MDA-MB-231 cells. This HIF-2α mediated reduction of lipid droplets was also observed in SUM149 cells. Silencing HIF-1α left the increased ECM degradation response to hypoxia, also observed in parental MDA-MB-231 cells, as well as increase of glycolysis under hypoxia largely intact. Silencing HIF-2α reduced this response, identifying the role of HIF-2α but not HIF-1α in this process in MDA-MB-231 cells. Interestingly, in SUM149 cells silencing HIF-1α, but not HIF-2α, reduced glycolysis in response to hypoxia.

Although ECM degradation increased with hypoxia, the invasion index was not altered in HIF-1α or HIF-2α silenced cells. Only double silenced MDA-MB-231 and SUM149 cells showed a significant decrease in invasion under normoxia with no further changes in response to hypoxia as evaluated in MDA-MB-231 cells. This reduction in invasion was not due to reduced proliferation in the double silenced cells, since there was no significant difference in proliferation in the MDA-MB-231 and SUM149 sublines when compared to parental or empty vector cells (submitted as Supplementary Data). Differences in the invasion of intact MDA-MB-231 sublines obtained using the MR compatible cell perfusion assay was validated with a conventional transwell assay. Both HIF-1α and HIF-2α are engaged in the hypoxia–mediated regulation of multiple genes involved in tumor growth and progression [19]. The role of HIF-1α in cancer is well studied [20], and the role of HIF-2α is increasingly being explored [21]. Here we investigated the role of silencing individual and both isoforms in two TNBC cell lines.

The role of HIFs in activating genes in the proteolytic cascade is well documented [22, 23]. In MDA-MB-231 cells, the proteolytic activity of matrix metalloproteinase (MMP) enzymes such as MMP-2, MMP-9 and MT1-MMP that augment invasion have frequently found to be upregulated in response to hypoxia [24, 25]. While HIF-1α is known to regulate proteolytic enzymes such as uPAR, cathepsins, MMP-1, MMP-2, MMP-9 in many cancer types [22, 26], HIF-2α has directly been involved in membrane bound MT1-MMP in von Hippel-Lindau renal cell carcinoma [27]. HIF-2α also appears to regulate MMP-2 levels in breast cancer. A recent study with human breast cancer specimens established a correlation between MMP-2 and HIF-2α expression [28]. HIF-2α expression was also significantly associated with lymph node metastasis [28].

The metastatic journey of cancer cells from the primary site to distant organs involves local invasion, intravasation of the tumor cells into the blood stream, survival of the circulating cells, extravasation at distant sites and colonization [29, 30]. Experimental metastasis was most profoundly affected in double silenced cells, followed by HIF-2α cells. HIF-1α silenced cells also displayed a significant decrease of metastasis although not as dramatic as the effects of HIF-2α and double silencing, suggesting that although invasion and ECM degradation abilities were not affected by HIF-1α silencing, these cells were less capable of establishing metastasis once injected into the bloodstream. HIF-1α plays an important role in angiogenesis [31] and silencing this pathway may have reduced the ability of cells to establish a vascular network and colonize at metastatic sites, even though the invasive phenotype was still present.

Several studies with TNBC have emphasized the role of HIF isoforms in tumorigenesis and metastasis [32–36]. In a study with MDA-MB-435 breast cancer cells, it was found that only HIF-1α was essential for hematogenous metastasis to the lungs, with no apparent role for HIF-2α, suggesting a differential role of HIF isoforms in the metastatic process [34].

The role of HIF-2α in breast cancer cells is being increasingly highlighted [28, 35–37]. In a study of 62 infiltrating ductal carcinomas, a significant correlation was observed between HIF-2α expression and lymph node metastasis, HER2/neu overexpression, and high vascular density [35]. HIF-2α is important in recruiting tumor-associated macrophages (TAM) in hypoxic regions in breast cancer [36]. A significant correlation was observed between high HIF-2α expression in TAM and high tumor vascularity and tumor grade [36]. Increased focal infiltration of macrophages into human breast tumors was directly associated with both angiogenesis and poor relapse free and overall survival [38]. Immunohistochemical studies involving breast cancer clinical samples also associated high levels of HIF-2α to tumor size, lymph node metastases, distant metastasis, and poor prognosis in patients with breast cancer [28], and to histology grade, Ki67 and ABCG2 expression [39]. However, none of the HIF-2α studies examined HIF-1α in the samples. In a study investigating the role of both HIF-1α and HIF-2α in breast cancer patient survival, HIF-2α but not HIF-1α was identified as an independent prognostic factor associated with reduced recurrence-free and breast cancer-specific survival [37]. Patients with HIF-2α positive cancers had an increased likelihood of distant recurrence. Our results are consistent with the importance of HIF-2α in invasion and metastasis. HIF-2α has been observed to persist throughout prolonged exposure to hypoxia, whereas HIF-1α accumulates initially and decays within a few hours [40, 41]. This may explain the dominant role of HIF-2α in some of the functional roles of the two isoforms investigated in this study.

We observed a significant increase of lipids and lipid droplets in response to hypoxia in MDA-MB-231 cells that considerably diminished with HIF-2α and double silencing, but not with HIF-1α silencing. This HIF-2α and double silencing mediated decrease of lipid droplets was confirmed in SUM149 cells. Lipids promote different aspects of cancer development by modulating growth, proliferation, and survival under oxidative and energy stress, supporting a high-glycolytic rate by promoting redox balance, and stimulating signaling pathways that lead to proliferation and invasion [42–45].

One mechanism by which HIF-2α modulates lipid droplets to sustain endoplasmic reticulum homeostasis under nutrient and oxygen deprivation was recently identified in clear-cell renal carcinoma [46]. In these studies, HIF-2α was observed to promote lipid storage through upregulation of the lipid droplet coat protein perilipin 2 (PLIN2) [46]. Consistent with our observations in TNBC, HIF-2α suppression resulted in a significant decrease of lipid droplets [46]. In another recent study, characterization of lipid droplets in nonmalignant and malignant breast cancer cells by Raman imaging revealed a marked increase in the number of lipid droplets with increased aggressiveness [47]. Remarkably, the content of these droplets in malignant breast cells was dominated by the arachidonic acid profile, unlike the triglycerides of oleic and linoleic acids that dominated the content of adipocytes [47]. Collectively, these results suggest that HIF-2α may also be implicated in the formation of prostaglandins and leukotrienes in cancers, further highlighting the potential importance of HIF-2α specific inhibitors in the treatment of TNBC, in addition to their current development in treating clear cell renal carcinoma (Clinical Trial No. NCT02293980). Our data also suggest that the lipid signal may provide a biomarker to noninvasively detect response to such inhibitors with ^1^H MRS.

Hypoxia has been observed to increase triglyceride accumulation in lipid droplets through increased Lipin 1 expression in a HIF-1α dependent mechanism, as shown in HeLaM cervical adenocarcinoma and Huh7 human hepatoblastoma cells [48]. We observed a significant reduction of Lipin 1 in HIF-2α silenced and DS cells under normoxic and hypoxic conditions. These results suggest a greater role of HIF-2α in regulating Lipin 1 in MDA-MB-231 cells. Although the HIF-2α and double isoform mediated changes in lipid droplets observed in MDA-MB-231 cells were recapitulated in SUM149 cells, Lipin 1 was not detectable in SUM149 cells. These results suggest that the role of the HIF-α isoforms and pathways in the lipid response to hypoxia may vary across cells lines, highlighting the importance of designing HIF inhibitors that act across different isoforms.

Cancer cells maintain high aerobic glycolytic rates and produce high levels of lactate [49] as observed by Warburg several decades ago (the Warburg effect). Increased serum LDH levels predict a poor outcome in a spectrum of neoplastic diseases [50]. LDHA is a key enzyme in the conversion of pyruvate to lactate. Upregulation of LDHA by cancer cells results in a predominantly glycolytic metabolism that reduces tumor dependence on the presence of oxygen. All the cells and sublines showed increased lactate in response to hypoxia, although basal lactate levels in the media decreased significantly in double silenced cells. In MDA-MB-231 cells, significantly lower lactate concentrations and higher glucose concentrations were also observed in HIF-2α (HIF-1α for SUM149) and double silenced cells relative to the corresponding empty vector cells under normoxic conditions and hypoxic conditions.

Single isoform silencing maintained the increased LDHA and lactate production response of MDA-MB-231 cells to hypoxia. Only double silenced cells showed reduced LDHA levels under normoxia and hypoxia. These results indicate that the presence of HIF-1α or HIF-2α can result in increased lactate levels, compensating for the silencing of the other in response to hypoxia. The reduction of lactate in medium of double silenced cells was reflected in the higher glucose concentrations in the medium due to reduced consumption of glucose. Although the role of HIF-1α in the induction of glycolytic enzymes such as glucose transporters (GLUT1 and GLUT3), hexokinases (HK1 and HK2) PFK-1, phosphoglycerate kinase 1 and LDH has been reported [3], the role of HIF-2α in regulating these enzymes has not been studied.

Understanding the divergent roles of HIF-α isoforms in cancer invasion, metastasis, and metabolism provides new insights into the role of hypoxia in these processes. By silencing HIF-α isoforms in TNBC cells, we have identified the importance of HIF-2α in mediating the invasive, lipid, and metabolic response of these cells to hypoxia in two cell lines. These studies support the expanded investigations of the role of HIF-2α in TNBC, and highlight the importance of developing HIF inhibitors that act across different isoforms in developing treatment strategies for TNBC.

## MATERIALS AND METHODS

### Cloning and generation of genetically modified MDA-MB-231 and SUM-149 sublines

The strategy for cloning, generation, and validation of HIF-1α and HIF-2α silenced breast cancer cells has been previously reported [17]. To establish MDA-MB-231 and SUM-149 cells silenced for both HIF-1α and HIF-2α expression (double silenced-DS cells), virions expressing HIF-2α shRNA were added to 231-HIF-1α shRNA or SUM-HIF-1α shRNA cells and downregulation was confirmed by quantitative real-time PCR (qRT-PCR) and immunobloting. The corresponding empty vectors for each HIF isoform and double silenced cells were characterized and showed a comparable hypoxic response to each parental cell lines.

### Cell proliferation

Briefly, parental, empty vector and modified sublines of MDA-MB-231 and SUM-149 breast cancer cells were plated at 2.5 × 10^5^ cells per well in a 6-well culture plate. Cellular proliferation was measured following trypsinization and staining with trypan blue, by counting cells at 24 h and 48 h using a hemocytometer. All experiments were performed in triplicate and the experiment was repeated thrice.

### Cells and cell culture conditions

MDA-MB-231 wild-type (231-WT) breast cancer cells (ATCC, Manassas, VA), and genetically engineered sublines transduced by lentivirus to stably express either HIF-1α shRNA (231-HIF-1α shRNA), HIF-2α-shRNA (231-HIF-2α shRNA) or both HIF-1α and HIF-2α shRNA (231-DS), and their empty vector counterparts (231-EV), were maintained in RPMI 1640 medium (Corning, Tewksbury, MA) supplemented with 10% fetal bovine serum (Sigma-Aldrich, St. Louis, MO). SUM149 wild-type (SUM-WT) breast cancer cells (Asterand, Inc., Detroit, MI) and genetically modified sublines expressing HIF-1α shRNA (SUM-HIF-1α shRNA), HIF-2α shRNA (SUM-HIF-1α shRNA) or both HIF-1α and HIF-2α shRNA (SUM-DS) were maintained in Dulbecco's modified Eagle's medium-F12 (1:1) (Corning) with 5% FBS, insulin (5 μg/ml), hydrocortisone (1 μg/ml). Hypoxic treatment of cells for 4–6 h was performed by placing the plates in a modular incubator chamber (Billups-Rothenberg, Del Mar, CA), flushed at 2 p.s.i. for 3 minutes with a gas mixture of 1% O_2_, 5%CO_2_, and N_2_ for the balance, or by treating cells with 200 μM of CoCl_2_ to chemically mimic hypoxia.

### Protein isolation and western blots

Total protein from transduced cells was extracted using RIPA lysis buffer containing a protease inhibitor. 100 μg total lysate were resolved on 7.5% SDS-PAGE. A mouse monoclonal antibody against HIF-1α (Novus Biologicals, Littleton, CO) at 1:1000 dilution, a rabbit polyclonal antibody against HIF-2α (Novus Biologicals) at 1:500 dilution, a rabbit polyclonal antibody against Lipin 1 (Cell signaling, Danvers, MA) at 1:1000 dilution, and a mouse monoclonal antibody against GAPDH (Sigma) at 1:10000 dilution were used. Appropriate horseradish peroxidase–conjugated secondary antibodies, either anti-mouse or anti-rabbit (GE Healthcare, Piscataway, NJ), were used at 1:2500 dilution. Immunoblots were developed using the SuperSignal West Pico chemiluminescent substrate kit (Thermo Scientific, Rockford, IL).

### MR data acquisition with MR compatible perfusion assay

Four days prior to the MR experiments, cells were seeded on Plastic Plus (Solohill Engineering Inc., Ann Arbor, MI) microcarrier beads at a cell density of 1.5 × 10^6^ cells per 0.5 ml of beads in non–cell culture petri-dishes (Labtec, Nunc, Denmark) and grown to approximately 70% confluency. Details of the perfusion system are previously described [51]. Briefly, adherently cancer cells grown on beads were layered above and below a chamber containing Matrigel^®^ at a concentration of 8.8 mg/ml as extracellular matrix (ECM), to determine the degradation of ECM by the cancer cells. The oxygen tension in the sample was kept at [O_2_] ≥ 20% during control experiments, and adjusted to [O_2_] ≤ 1% in experiments studying the effects of hypoxia on the invasion and metabolism of cancer cells. A layer of perfluorocarbon doped alginate beads were interspersed within the layers of cancer cells to monitor the oxygen tension in the sample using ^19^F MR relaxometry. The temperature was maintained at 37°C and the pH at 7.30 ± 0.15 for all MR experiments.

The following MR data were acquired on a 9.4 T MR spectrometer (Bruker, Billerica, MA) every 12 h over a period of 2 days. Proton MR imaging was performed to evaluate the overall sample preparation, to visualize the geometry of the ECM, and to detect changes in the integrity of the ECM due to invasion and degradation by cancer cells. Degradation of ECM by cancer cells was determined at the 24 h time point relative to the initial time point from the proton images. The extent of ECM degradation was estimated by drawing a region of interest (ROI) around the ECM gel region using NIH ImageJ software. The degradation index was defined as (ROI_*t* = 0_ –ROI_*t* = 24_)/ROI_*t* = 0_. One dimensional (1D) ^1^H MR profiles of intracellular water were acquired along the length of the sample to quantify the number of cells invading the ECM and obtain an invasion index as described previously [51]. Diffusion weighted (DW) 1D ^1^HMR spectra were acquired using lactate-editing to quantify the contribution of lactate and lipids. DW 1D ^1^H MR spectra obtained without water suppression were used to determine cell proliferation because the increase of slow-diffusing water, which represents intracellular water, was directly proportional to the number of cells. Localized DW 1D ^1^H chemical shift imaging (CSI) spectra with and without water suppression were acquired every 24 h to obtain metabolic information from 310-μm-thick slices along the z-axis of the sample. The oxygen tension was obtained from slice-selective 1D ^19^F inversion recovery T_1_ measurements of the perfluorocarbon beads. The MR data acquisition, processing, and analysis were performed as previously described [51]. Experiments with 231-WT (*n* = 7 for normoxia and *n* = 6 for hypoxia; 231-HIF-1α shRNA (*n* = 7 for normoxia and *n* = 5 for hypoxia); 231-HIF-2α shRNA (*n* = 3 for normoxia and *n* = 3 for hypoxia); 231-DS (*n* = 5 for normoxia and *n* = 4 for hypoxia) were carried out.

### Transwell chamber invasion assay

Cell invasion was measured using Corning Biocoat Matrigel Invasion chambers incorporating polyethylene terephthalate track-etched membranes with a pore size of 8.0 μm (Corning, Tewksbury, MA). Twenty five thousand cells were plated in serum free medium in the upper chamber and 5% FBS containing medium was placed in the lower chamber to act as a chemoattractant. At the end of 20 h, cells that invaded through the membrane were stained with a solution of 0.2% crystal violet in ethanol. Cell numbers from at least 3 fields of view (FOV) per membrane were counted. Each experiment was performed in duplicate with two separate experiments performed per cell line.

### Establishing experimental metastasis *in vivo* and determining metastatic burden

Approximately 10^6^ 231-WT cells or genetically modified sub lines were injected intravenously in 200 μl of Hanks balanced salt supplement (HBSS, Sigma-Aldrich) through the tail vein of age-matched severe combined immunodeficient mice (SCID, Charles River, Wilmington, MA) with five animals in each group. Five weeks post injection, mice were sacrificed, and the lungs inflated, formalin fixed, and paraffin embedded for sectioning. Hematoxylin and eosin (H&E) staining was performed on 5 μm sections from paraffin embedded blocks. High-resolution image scans of H&E stained lung tissue slides were performed on an ImageScope digital scanner (Aperio technologies Inc., CA, USA). Metastatic burden was calculated as: (area of the metastatic foci/total area of the lung) x100. All animal experiments were performed in accordance with protocols approved by the Johns Hopkins University Institutional Animal Care and Use Committee.

### Nile red staining for lipids

Cells were grown on glass chamber slides (Thermo Fisher, Rochester, NY, USA) to 60% to 70% confluence, washed with PBS, and fixed with 4% (w/v) paraformaldehyde. Cells were washed with PBS and incubated at a 1:1000 dilution in PBS of a 1 mg/ml stock solution of Nile Red (Sigma-Aldrich) in acetone for 10 min at room temperature. Cell nuclei were counterstained and mounted with ProLong Gold Antifade reagent (Life Technologies, Grand Island, NY). A 40X LUMPLFL water immersion objective on an Olympus FV1000MPE multiphoton microscopy system (Olympus, Center Valley, PA) with a 690–1040 nm tunable MaiTai DeepSea femtosecond laser (Spectra-Physics, Santa Clara, CA) was used to acquire three dimensional (3D) image stacks from various fields of view in the sample. DAPI stained nuclei and Nile Red stained lipid droplets were simultaneously illuminated with incident laser light of 810 nm and detected at 455 nm (blue channel, DAPI) and 640 nm (red channel, Nile Red) respectively. The number and size of lipid droplets per cell were quantified using in- house software. Approximately 20–40 cells per field of view from at least six different fields of view were analyzed for each cell line.

### Lactate and glucose measurement

To measure extracellular lactate concentration in the medium approximately 3 × 10^5^ cells were plated overnight. One ml of medium was collected from cells maintained under normoxia or under hypoxia (1% O_2_ treatment) for 72 h. Lactate and glucose measurements were carried out using a Radiometer ABL 700 series blood-glucose analyzer (Radiometer, Copenhagen, Denmark). For lactate dehydrogenase (LDH) mRNA studies, total RNA was isolated and cDNA was prepared from 231-EV cells and HIF-α modified sublines using standard protocols. Data from empty vector cells corresponding to their HIF-α silenced cells counterparts were pooled together for comparison with HIF-α silenced cells. qRT-PCR for LDHA mRNA expression was carried out using the forward primer, ACCCAGTTTCCACCATGATT and the reverse primer CCCAAAATGCAAGGAACACT. Three independent experiments were performed and values are expressed as mean ± SEM.

### Statistical analysis

Statistical significance was evaluated using the Mann-Whitney *U* test with *p* values < 0.05 considered statistically significant, unless otherwise stated.

## SUPPLEMENTARY FIGURE



## Abbreviations

CSI: chemical shift imaging
DW: diffusion-weighted
ECM: extracellular matrix
ER: estrogen receptor
GLUT: glucose transporter
HIF: hypoxia inducible factor
HK: hexokinase
LDH: lactate dehydrogenase
MMP: matrix metalloproteinase
MR: magnetic resonance
VHL: von Hippel-Lindau
PR: progesterone receptor
qRT-PCR: quantitative real-time PCR
SCID: severe combined immunodeficient mice
shRNA: short-hairpin RNA
TAM: Tumor associated macrophage
TNBC: triple negative breast cancer
