# Supplementary material for: HIF isoforms have divergent effects on invasion, metastasis, metabolism and formation of lipid droplets
Source: Oncotarget. 2015 Jul 20;6(29):28104–19. doi: 10.18632/oncotarget.4612 (PMC4695047; doi:10.18632/oncotarget.4612)
Supplement: Supplementary file 1 [file oncotarget-06-28104-s001.pdf]

SUPPLEMENTARY FIGURE

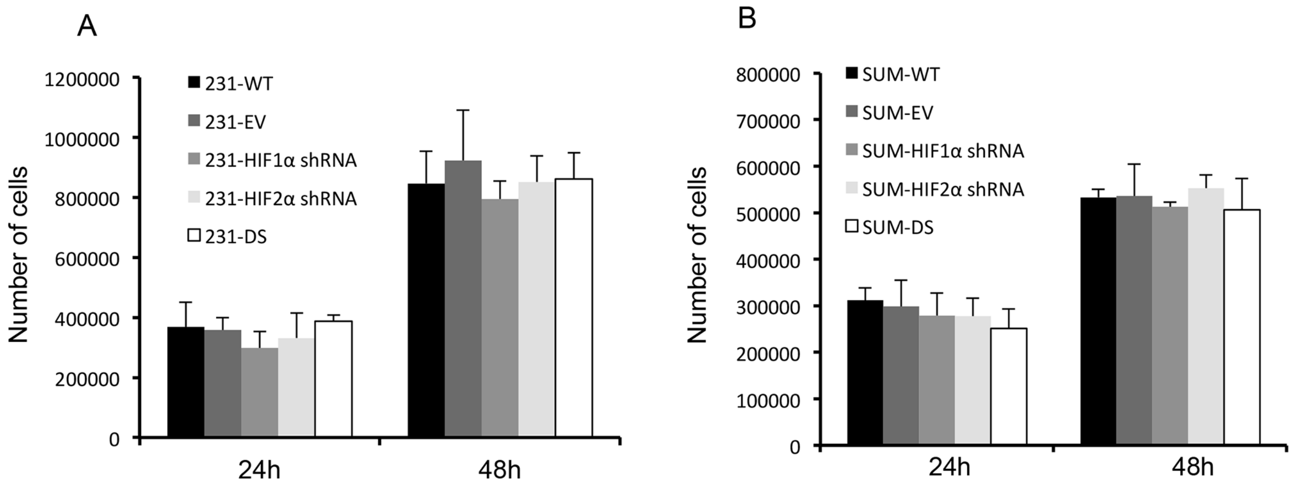

Supplementary Figure S1: Cellular proliferation measured in A. MDA-MB-231 and B. SUM149 sublines following trypsinization and staining with trypan blue, by counting cells at 24 h and 48 h using a hemocytometer. Values represent Mean ± SE, *n* = 3.
